# Value of Echocardiography in the Treatment of Patients With Acute Heart Failure

**DOI:** 10.3389/fcvm.2021.740439

**Published:** 2021-11-15

**Authors:** Masaki Izumo

**Affiliations:** Division of Cardiology, Department of Internal Medicine, St. Marianna University School of Medicine, Kawasaki, Japan

**Keywords:** heart failure, echocardiography, acute heart failure, diagnosis, prognosis

## Abstract

Heart failure (HF) is a burden in pandemic medicine resulting in high mortality and morbidity. Because acute HF is a life-threatening event, its diagnosis and choice of optimal treatment are important to improve outcomes. Furthermore, understanding the cause and hemodynamics of acute HF is important in selecting the optimal treatment for these patients. Echocardiography is widely used in daily clinical practice because of its non-invasive nature and excellent portability to understand cardiac function and hemodynamics. Echocardiography is highly recommended by guidelines in the practice of HF, but evidence is limited. In this review, I would like to share clinical value of echocardiography in the treatment of patients with acute HF and discuss the usefulness of echocardiography.

## Introduction

Heart failure (HF) places a burden on the healthcare system, which has increased during the pandemic, and resulted in high mortality and morbidity (1–5). More than 1 million people in the US and Europe are hospitalized annually for acute decompensated HF (ADHF) (1–5). Echocardiography is widely used for the diagnosis and during the management of HF and is used in various situations such as evaluation of cardiac function and hemodynamics, guiding treatment decisions, and assessing the efficacy of treatment. Performing echocardiography, as an initial investigation, is a class I recommendation in the HF guidelines of various countries (6–10). Furthermore, echocardiography is considered to be useful for guiding treatment of ADHF because it is the only imaging modality available at the patient's bedside that can be evaluated in real time with minimal invasion. However, while there is much evidence that echocardiography is useful in chronic HF, there is little evidence to suggest that echocardiography is useful in the management of ADHF (10, 11). Despite guideline recommendations, echocardiography was infrequently performed in patients with HF in routine clinical practice (10, 12, 13). Thus, it is unclear whether and how echocardiography can be used in patients with ADHF and what phenotypic echocardiographic findings can be used to select patients for intervention. In this review, I would like to share the clinical value of echocardiography and discuss its usefulness in the treatment of patients with acute HF.

## Etiology of HF

**Figure 1:**
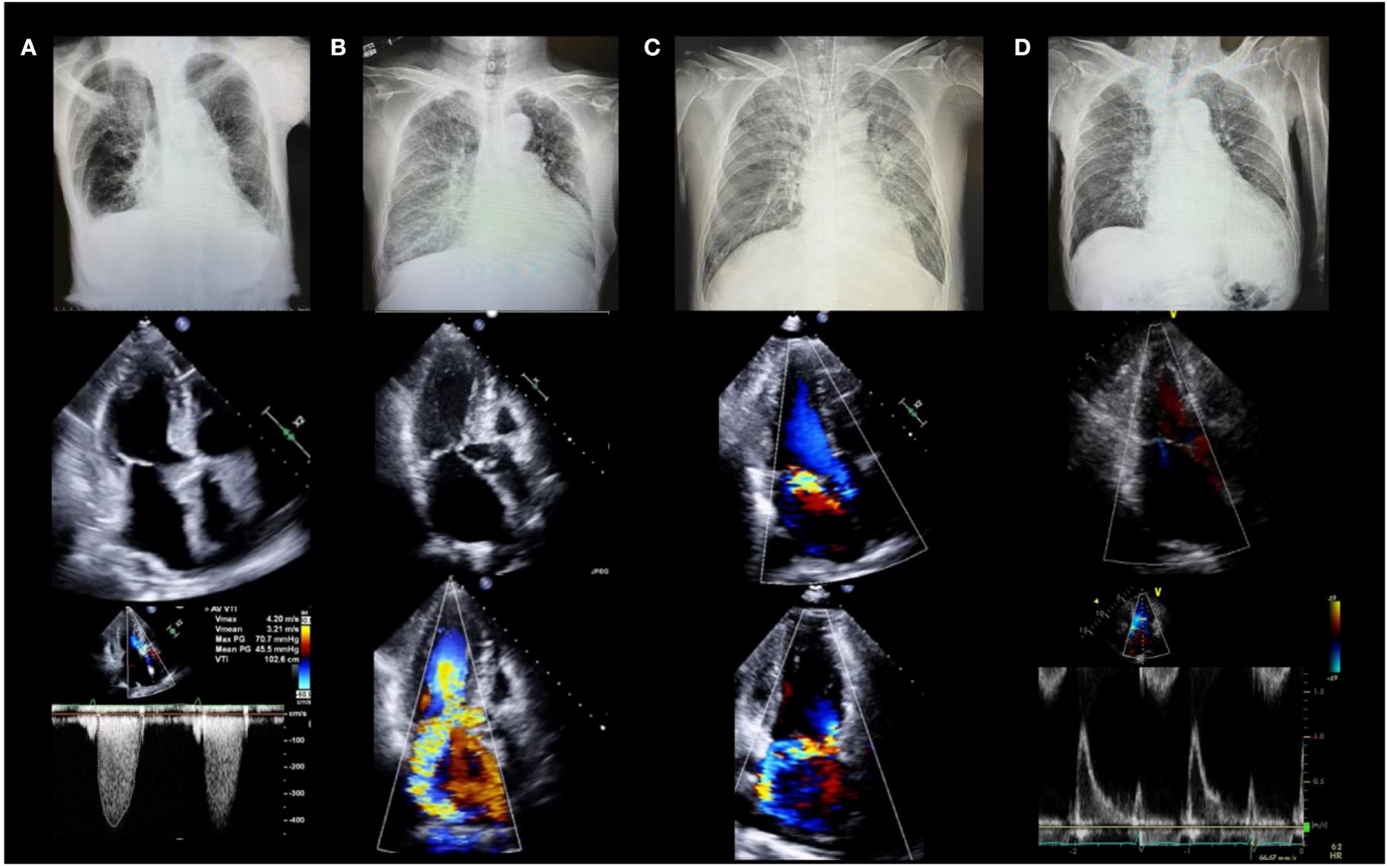
Four cases of acute heart failure. All patients had dyspnea and acute pulmonary edema with systolic blood pressure >140 mm Hg (clinical scenario 1) and preserved left ventricular ejection fraction. **(A)** 88-year-old female with severe aortic stenosis. Lower panel shows peak velocity of 4.2 m/s and mean pressure gradient of 45.5 mmHg. **(B)** 78-year-old female with hypertrophic cardiomyopathy. Middle and lower panels show severe mitral regurgitation due to systolic anterior motion of the mitral valve. **(C)** 64-year-old male with acute mitral regurgitation. Middle and lower panels show severe mitral regurgitation due to posterior leaflet prolapse without left atrial enlargement. **(D)** 78-year-old female with heart failure with preserved ejection fraction. Middle and lower echocardiographic images show severe left ventricular diastolic dysfunction without structural abnormalities.

Determining the etiology of HF is important for selecting the appropriate management. The incidence of valvular disease is increasing in an aging society. According to the Euro Heart Survey, valvular heart disease was present in one-third of the patients with acute HF in Europe. Approximately 10% of patients with acute HF have aortic stenosis, and more than 40% have MR (14). Acute HF with valvular disease is not uncommon, and echocardiography is the only modality that can be used to diagnose valvular disease in an emergency setting. Herein, we present chest radiographs and echocardiography findings for four patients with HF who developed acute pulmonary edema (Figure 1). The etiologies of HF with preserved ejection fraction (EF) were different in each case: Patient A had aortic stenosis, B had hypertrophic obstructive cardiomyopathy, C had acute MR, and D had HF without structural abnormalities. It is important to determine the cause of the HF when deciding on an appropriate treatment. Vasodilators and diuretics are the mainstay of treatment; however, vasodilators should be avoided in cases similar to those of patients A and B, and their use is recommended in cases similar to those of C and D.

## Evaluation of Hemodynamics

**Figure 2:**
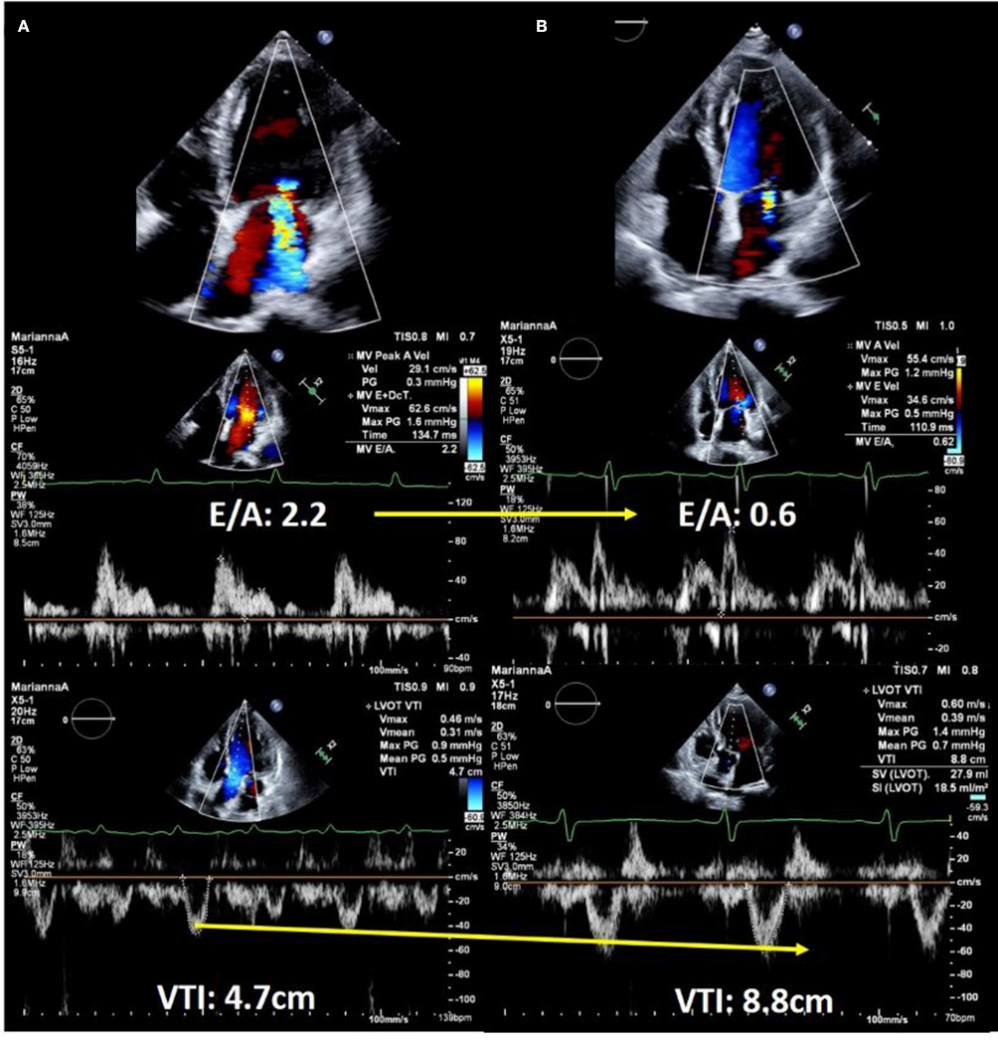
Echocardiographic images of a 58-year-old man with dilated cardiomyopathy showing response to heart failure therapy. **(A)** On admission. Upper panel shows severe secondary mitral regurgitation with dilated left ventricle. Middle panel shows restrictive left ventricular inflow pattern and velocity time integral of only 4.7 cm, which suggested low forward cardiac output. **(B)** After 14 days of hospitalization. Color Doppler echocardiography shows a significant reduction in mitral regurgitation (upper panel). Doppler echocardiography showed decreased left ventricular filling pressure (middle panel) and significantly improved forward cardiac output (lower panel).

Echocardiography is performed for the evaluation of hemodynamics and estimation of left ventricular (LV) filling pressures as well as assessment of structural disorders in patients with acute HF (10, 15–18). Forrester classification using pulmonary wedge pressure and cardiac index is useful for evaluating hemodynamics and determining treatment options for acute HF (19, 20). However, this requires an invasive procedure i.e., Swan-Ganz catheterization, and is, therefore, used less frequently. We can estimate the wedge pressure using echocardiography as well. Based on the American Society of Echocardiography guidelines, interpretation of the LV inflow pattern is simple and can be used to estimate the LV filling pressure. E/A of 2 or greater indicates that the LV filling pressure is increased (16). E/A is a simple and highly reproducible test that is useful in an emergency setting. However, during the 0.8 ~2 of E/A, patients with HFpEF and/or atrial fibrillation often suffer from an elevated left ventricular filling pressure. Making a diagnosis with an E/e' ≥13 on tissue Doppler imaging, a tricuspid flow velocity ≥2.8 m/s, and BNP may be helpful in daily clinical practice (21). Measurement of the velocity time integral (VTI) at the LV outflow tract (LVOT) is a simple and useful method of estimating the forward stroke volume and cardiac output (22–25). LVOT VTI is useful for estimating not only the cardiac output but also the prognosis in HF with either preserved or reduced EF (25, 26). Changes in LV inflow pattern and LVOT VTI are useful for assessing treatment response. A case of acute HF with reduced EF is shown in Figure 2. It describes the case of a 58-year-old male with non-ischemic cardiomyopathy. Echocardiography showed severely impaired LVEF and severe secondary MR. On admission, LV inflow had a restrictive pattern and VTI was very low. The patient was determined to have Forrester classification IV HF, and he was started on vasopressor support. Repeat echocardiography was performed to confirm the response to treatment. The LV inflow pattern gradually improved, and LVOT VTI increased. Secondary MR improved dramatically in this case, and we could confirm the impact of HF treatment on echocardiography. Estimation of peak systolic pulmonary arterial pressure (SPAP) is important in clinical practice (27–29). Most patients with ADHF had either passive or mixed pulmonary hypertension (30). There are two forms of pulmonary hypertension due to left-sided heart disease: post-capillary pulmonary hypertension, which is caused by the passive propagation of elevated left atrial or pulmonary venous pressures into the pulmonary arteries, and pre-capillary pulmonary hypertension, which is caused by pulmonary artery remodeling and further pulmonary artery pressure elevation. Combined pre- and post-capillary pulmonary hypertension has been shown to have a worse prognosis than passive pulmonary hypertension. It has been suggested that therapeutic intervention is necessary for patients with reactive pulmonary hypertension, and it is critical to consider this diagnosis and subsequent management strategies for patients with acute heart failure. SPAP is estimated from the peak velocity of the tricuspid regurgitation jet and right atrial pressure (28, 30). A correlation between echocardiographic and invasive assessment of SPAP in patients with acute HF has been reported, and echocardiography is used to assess SPAP in clinical practice (27). A Japanese multicenter study reported that changes in tricuspid regurgitation peak gradient (TRPG) had additive value in predicting the prognosis of worsening renal failure in acute HF. Worsening renal failure with increased TRPG levels is associated with a poor prognosis (31).

## Assessment of Cardiac Function

Although there are various parameters for assessing cardiac function, left ventricular EF (LVEF) is simple and is one of the most commonly used parameters in clinical practice. A recent US study of Medicare recipients reported that ~40% of patients with ADHF did not have an echocardiographic EF assessment after diagnosis of HF (12). In that study, the proportion of patients who underwent LVEF evaluation increased over time, but still a lower proportion of women, blacks, older patients, and outpatients underwent echocardiography. In contrast, in the US registry study, the percentage of patients who underwent echocardiographic LVEF assessment was significantly higher when LVEF assessment was considered as one of the endpoints (32, 33). In the ADHERE registry, which entered 160,000 HF patients admitted to 285 hospitals in the United States between January 2002 and December 2004, the percentage of patients who evaluated echocardiographic LVEF increased from 82.5 to 88.9% over 3 years (32). In the OPTIMIZE-HF registry, which was designed to test whether performing guidelines would improve patient care in ADHF, performance of evidence-based practice reportedly improved over time, and the percentage of patients who underwent LVEF assessment increased from 89 to 92% over a 21 month period. Fonarow et al. (33) Hospitals that received achievement award in the American Heart Association program for improving outcomes in cardiovascular diseases, including HF, have higher rates of echocardiographic LVEF assessment compared to hospitals that have not received the award (34). Currently, ~50% of the patients with HF have preserved EF (35, 36). Adamopoulos et al. reported that the prognostic value of LVEF assessed at admission increased when combined with mean arterial pressure of patients with acute HF (37). Furthermore, the effect of medication for HF differs based on LVEF. The prognostic value of response to β-blockers and angiotensin-converting enzyme inhibitor/angiotensin receptor blocker has been demonstrated in many studies in patients with reduced EF, but not in patients with preserved LVEF (36). Echocardiography is less invasive and expensive than computed tomography or magnetic resonance imaging, and it can be performed repeatedly for patients with HF. LVEF changes with treatment, the so-called reverse remodeling; therefore, changes in LVEF can predict the prognosis. Many studies have reported that HF with recovered EF has a better prognosis (38, 39). Echocardiographic follow-up can be used to estimate the prognosis of patients with HF. Since LVEF reflects not only LV contractility but also other factors, such as heart rate, blood pressure, and volume overload, it is necessary to consider these factors when evaluating contractility using LVEF. When evaluating mitral and aortic valve insufficiency using LVEF, it is especially important to be aware of these factors to ensure that contractility is not overestimated. LVEF also overestimates contractility when LV wall thickening is present (hypertensive heart disease, hypertrophic cardiomyopathy, etc.); in such cases, mid-wall fractional shortening or other calculations are preferred.

Acute right heart failure is a complex disease that is difficult to diagnose and manage in daily clinical practice. Ventricular interactions also affect the structure and function of each ventricle, as well as ventricular hemodynamics and events. It is important to evaluate not only the LV in acute heart failure, but also the right ventricular geometry, function, and pulmonary artery coupling using echocardiography (40). Assessing the inferior vena cava diameter and respiratory collapse helps in estimating the right atrial pressure, although the quantitative method (pulmonary artery systolic pressure—tricuspid pressure) is more reliable than the more invasive measurement. An apical or subcostal view is used to assess the RV size, LV-RV balance, and RV sphericity.

## Additive Value of Lung Ultrasound

A recent meta-analysis reported that B-profiles identified using lung ultrasound were more accurate for detecting cardiogenic causes of dyspnea than pleural effusion and TTE (41–43). The AHF Group of the European Society of Cardiology has stated that for patients with dyspnea and shock, lung ultrasound can be easily and rapidly interpreted, and it is necessary to perform lung ultrasound, in addition to transthoracic echocardiography, in an emergent setting for patients with suspected acute heart failure (44).

## Conclusions

Echocardiography can reveal structural abnormalities, and assess cardiac function and hemodynamics. Although evidence is limited, echocardiography can provide important information for the diagnosis and treatment of acute HF. In addition, national guidelines recommend echocardiography in acute HF, and I believe that echocardiography should be used for the management patients with acute HF in the clinical setting.

## Author Contributions

The author confirms being the sole contributor of this work and has approved it for publication.

## Conflict of Interest

The author declares that the research was conducted in the absence of any commercial or financial relationships that could be construed as a potential conflict of interest.

## Publisher's Note

All claims expressed in this article are solely those of the authors and do not necessarily represent those of their affiliated organizations, or those of the publisher, the editors and the reviewers. Any product that may be evaluated in this article, or claim that may be made by its manufacturer, is not guaranteed or endorsed by the publisher.
